# The role of depression in the association between physiotherapy frequency and duration and outcomes after hip fracture surgery: secondary analysis of the physiotherapy hip fracture sprint audit

**DOI:** 10.1007/s41999-023-00808-8

**Published:** 2023-06-17

**Authors:** Rhian Milton-Cole, Matthew D. L. O’Connell, Katie Jane Sheehan, Salma Ayis

**Affiliations:** Department of Population Health Sciences, School of Population Health and Environmental Sciences, King’s College London, 4th Floor Addison House, Guy’s Campus, London, SE1 1UL UK

**Keywords:** Hip fracture, Depression, Rehabilitation, Older adults

## Abstract

**Purpose:**

It is not known whether the association between the frequency and duration of physiotherapy and patient outcomes varies for those with and without depression. This study aims to evaluate whether the associations between the frequency and duration of physiotherapy after hip fracture surgery and discharge home, surviving at 30 days post-admission, and being readmitted 30 days post discharge vary by depression diagnosis.

**Methods:**

Data were from 5005 adults aged 60 and over included in the UK Physiotherapy Hip Fracture Sprint Audit who had undergone surgery for a nonpathological first hip fracture. Logistic regression models were used to estimate the unadjusted and adjusted odds ratios and their 95% confidence intervals for the associations between physiotherapy frequency and duration and outcomes.

**Results:**

Physiotherapy frequency and duration were comparable between patients with and without depression (42.1% and 44.6%). The average adjusted odds for a 30-min increase in physiotherapy duration for those with and without depression for discharge home were 1.05 (95% CI 0.85–1.29) vs 1.16 (95% CI 1.05–1.28, interaction *p* = 0.36), for 30-day survival were 1.26 (95% CI 1.06–1.50) vs 1.11 (95% CI 1.05–1.17, interaction *p* = 0.45) and for readmission were 0.89 (95% CI 0.81–0.98) vs 0.97 (95% CI 0.93–1.00, interaction *p* = 0.09). None of the interaction tests reached formal significance, but the readmission models were close (*p* = 0.09).

**Conclusion:**

Results suggest physiotherapy duration may be negatively associated with readmission in those with depression but not those without depression, while no clear difference in the other outcomes was noted.

**Supplementary Information:**

The online version contains supplementary material available at 10.1007/s41999-023-00808-8.

## Key summary points

**Aim:**

The aim of this paper is to estimate the associations between the frequency and duration of physiotherapy after hip fracture surgery and discharge home, surviving at 30 days post-admission, and being readmitted 30-days post-discharge by depression diagnosis.

**Findings:**

The associations between physiotherapy frequency and duration and outcomes between groups with and without depression suggest some differences in the associations. There was no evidence of a significant formal interaction of a depression diagnosis in the association between physiotherapy frequency and duration and outcomes, but the test for the readmission-duration model was close.

**Message:**

Results suggest physiotherapy duration may be associated with readmission in those with depression but not those without depression, but no clear difference in the other outcomes.

**Supplementary Information:**

The online version contains supplementary material available at 10.1007/s41999-023-00808-8.

## Background

Hip fractures are a major cause of morbidity and mortality and often lead to poor outcomes such as a high chance of moving to a residential/nursing home and being readmitted to a hospital [1–4]. It is well established that physiotherapy rehabilitation after hip fracture surgery can reduce the risk of these poor outcomes [5]. Indeed, the Chartered Society of Physiotherapy (CSP) set care standards for Hip Fractures based on data from the Physiotherapy Hip Fracture Sprint Audit. These standards set quality guidelines all patients in the UK should receive [6]. They recommend: (1) hip fracture patients receive daily physiotherapy a minimum total of two hours in the first-week post-surgery; (2) physiotherapists must assess all hip fracture patients within a day of their surgery and (3) patients should be mobilised within a day of their surgery [6]. In addition, these CSP care standards were recently supported by a secondary analysis which showed in a hip fracture population, patients’ odds of being discharged home, surviving 30-days post-admission, and being readmitted 30-days post-discharge were improved with each 1-day increase in frequency and 30-min increase in the duration of physiotherapy received in the first postoperative week [7].

We do not yet know whether the association between rehabilitation and outcomes is consistent across subgroups of the hip fracture population. One such subgroup is patients with depression. Indeed, patients with both hip fracture and depression often have delayed and complicated hip fracture recoveries with many unable to return to baseline functioning [8–10]. Previous literature has shown older adults with depression or depressive symptoms are more likely to be re-admitted at 30 days following orthopaedic surgery [11, 12] and have higher rates of 30-day mortality after surgery [13–15]. It is not known whether physiotherapy frequency and duration vary for those with and without depression, nor whether any variation may lead to differences in these poor outcomes. Therefore, the aim of this paper is to estimate the associations between the frequency and duration of physiotherapy after hip fracture surgery and discharge home, surviving at 30 days post-admission, and being readmitted 30-days post-discharge by depression diagnosis.

## Methods

### Dataset

The dataset is taken from the Physiotherapy Hip Fracture Sprint Audit (PHFSA), a collaborative audit between the Royal College of Physicians’ (RCP’s), National Hip Fracture Database (NHFD) and the Chartered Society of Physiotherapy (CSP) [16]. The audit collected data in 2017 to gather information on patients surgically treated for a hip fracture in May or June 2017 [17] for all patients 60 years of age or older who underwent surgery for hip fracture in England and Wales. This dataset was then linked to the Hospital Episode Statistics for England [18] and the Patient Episode Database for Wales [19] for additional data including comorbidities, mortality, and readmission [20]. Further detail on the linkage of datasets, data cleaning and validation are reported elsewhere [20].

### Participants

Patients aged 60 years or older who had undergone surgery for their first hip fracture in England and Wales and whose data was submitted as part of the PHFSA were included. We excluded 378 patients with potentially pathological hip fractures by identifying patients with at least one hospitalisation record with cancer that commonly metastasises to bone (ICD-10 code: C34, C50, C61, C64, C65, C78, C79, C80, C90) and/or Paget’s disease ICD-10 code: M88). The technical report specifying the development of the analytical dataset is described in the supplementary file of a previous paper [20].

### Variables

#### Exposures

Exposures included frequency and duration of physiotherapy received in the first postoperative week. Frequency was defined by physiotherapy on 0–7 days of a possible 7 in the first postoperative week, and weekly duration was defined by 30-min increments ranging from 0–511 min across the first post-operative week.

#### Outcomes

Binary outcomes included discharged home (among those admitted from home), survival at 30-days post-admission, and readmission up to 30 days post-discharge.

#### Confounders

Potential confounders included age, sex (female, male), prefracture residence defined as nursing/residential care or own home/sheltered housing (not included for discharge home analysis), fracture type (Intracapsular, Intertrochanteric/Subtrochanteric), ambulation prior to hip fracture (indoors and outdoors, indoor only, no functional mobility), the timing of surgery defined as within target time of 36-h to surgery or not within a target time of 36-h to surgery, the timing of first mobilisation defined as within target time of day of surgery/day after surgery or not within a target time of day of surgery/day after surgery and a number of comorbidities [21–25]. These variables were included in the adjusted stratified and interaction models.

#### Depression

ICD-10 diagnosis codes of F20.4, F32, F33, F34 and F43 were used to identify those with and without a diagnosis of depression [26]. These codes were listed if patients presented with depression during the hip fracture hospitalisation or in the year prior to this hospitalisation.

When used, ‘patients (with a hip fracture and) with depression’ is defined as patients aged 60 or over with a hip fracture and a diagnosis of depression during the hip fracture hospitalisation or in the year prior to this hospitalisation.

### Sample size

We retrospectively calculated the sample size needed to allow us to detect statistically significant results for our stratified and interaction models although the full dataset was used in the analyses [27]. We used the parameter estimates from our regression models in the calculations and adjusted our calculations for the sample size needed for unequal groups due to the number of people with a diagnosis of depression being lower than the number of those without depression [28]. The range of Odds Ratios found across all our stratified and interaction models ranged from 0.90–1.30 (Tables 3 and 4). Using these ratios to retrospectively estimate our power calculations, we determined that to detect Odds Ratios within this range with 80% power we would need a sample size of 540 for each group [28]. After considering the sample size difference between those with and those without depression, we estimated the unequal sample sizes. To maintain 80% power in the group with a diagnosis of depression the sample size needed was 304, while for the group without a diagnosis of depression, the sample size needed was 2431.

### Statistical analysis

Patients with complete data for the exposure, outcome and confounding variables were used in the main analysis. Differences between patients with and without complete outcome and exposure data are presented in Online Resource 1. Patient characteristics are presented by the presence and absence of a diagnosis of depression and by the exposure variables of frequency of physiotherapy (0–5, 6–7 days) and duration (< 2 h, ≥ 2 h) in the first postoperative week. Categorical characteristics are presented as frequencies and percentages and continuous characteristics are presented as median and interquartile range (IQR). Chi-square test (categorical) and Mann–Whitney *U* test (continuous) were used to compare distributions across exposure groups for those with and without a diagnosis of depression.

Logistic regression models were used to estimate the unadjusted odds ratios and odds ratios adjusted for potential confounding variables and their 95% confidence intervals (CI) for the association between frequency (1-day increment) and duration (30-min increment) and being discharged home, surviving at 30-days post admission, and readmission at 30-days post-discharge. For the analysis of discharge home, we selected a subset of patients whose prefracture residence was their own home/sheltered housing and who were alive on discharge. To assess the influence of a depression diagnosis on the associations between physiotherapy frequency and duration and patient outcomes, logistic regression models may be used to formally test the interaction; first we examined the full sample for interactions. The interaction odds ratios reflect the difference in the association between physiotherapy frequency and durations’ interaction by depression diagnosis for the different outcomes. Due to the small and imbalanced sample sizes of the groups with and without depression and the risk of underpowered interaction models (the retrospective power calculations were completed after the analyses had been conducted), we ran logistic regression models for each of the samples with a diagnosis of depression and without, independently [29].

Multiple imputations by chained equations were used to assess the sensitivity of complete case analyses to the influence of missing data in exposure, confounding, and outcome variables [30]. Missing data for the outcome discharge home, was unlikely missing at random as some sites had greater access to inpatient rehabilitation than others therefore, we only adopted a complete case analysis for this outcome [31]. All analyses were completed in Stata 16.1 [32].

**Fig. 1:**
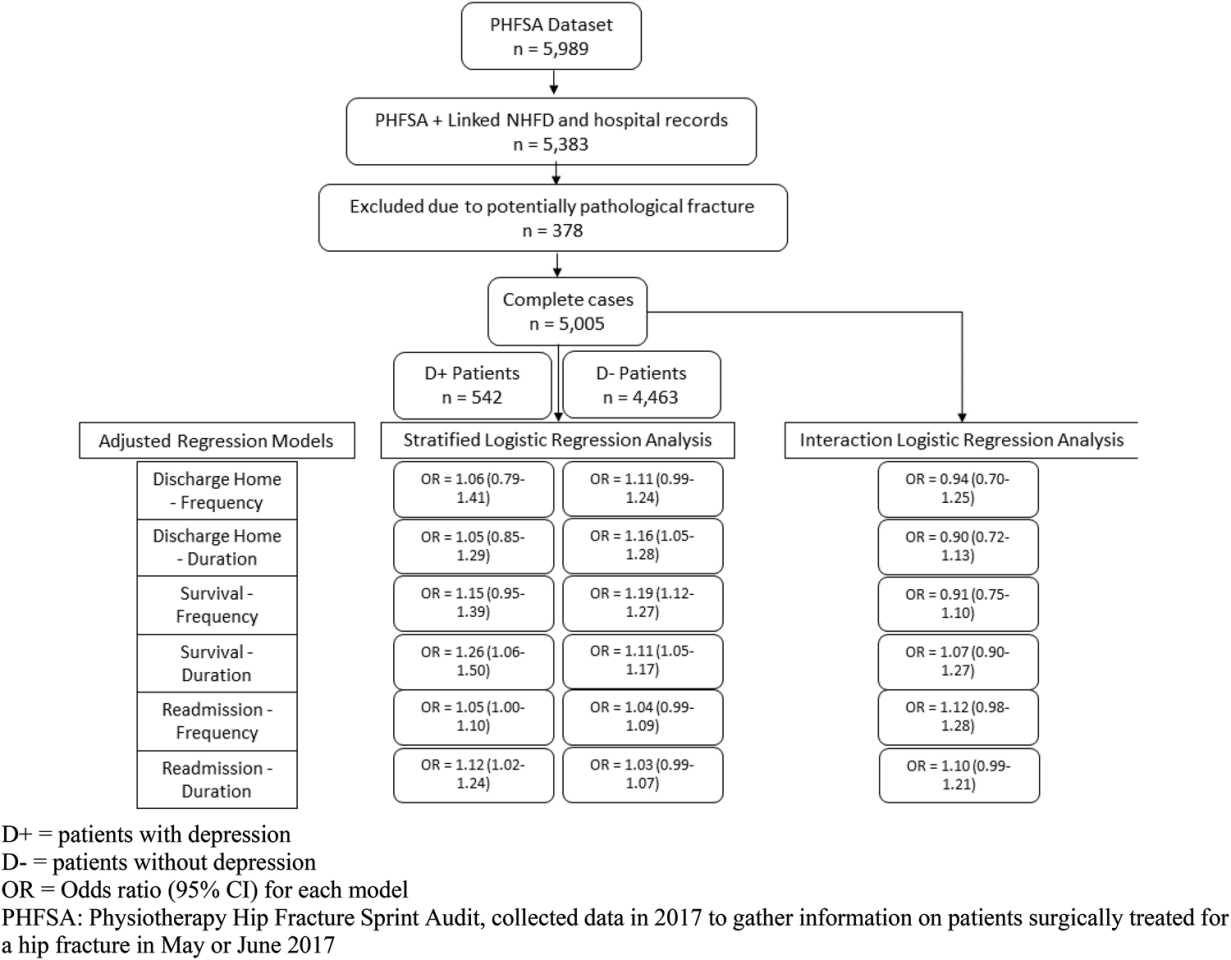
Diagram of the sample selection process, each statistical model and the adjusted models results

Figure 1 details the sample selection process, the statistical procedures and the main results obtained.

## Results

### Data and patient characteristics

Among 5989 patients included in the PHFSA, 5383 had linked NHFD and hospital records (inclusive of depression diagnosis code). Of the 5383, 378 patients had potentially pathological fractures and were excluded, leaving a sample of 5005 patients for analysis. Of these 5005, a diagnosis of depression was present in 542 (10.8%) patients. Overall, the median age of those with depression was 82 (IQR 76–88) and the majority were female (75.6%). In those without depression, the median age was 84 (IQR 78–89) and the majority were female (72.8%) (Table 1). The proportion admitted from home for those with depression was 75% and 82.2% for those without depression. The proportion of those with depression with independent indoor and outdoor mobility pre-fracture was 64.1% and 74.6% for those without depression (Table 1). Among those with depression, 228 (42.1%) received ≥ 2 h of physiotherapy and 91 (16.8%) received physiotherapy on 6–7 of a possible 7 days. Among those without depression, 1992 (44.6%) received ≥ 2 h of physiotherapy and 875 (19.6%) received physiotherapy on 6–7 of a possible 7 days (Table 2).

**Table 1:** Characteristics of 5005 patients surgically treated for non-pathological hip fracture by depression diagnosis and Frequency of physiotherapy

		Depression			No depression		
		Frequency			Frequency		
		All	0–5 days	6–7 days	All	0–5 days	6–7 days
		*n* = 542	*n* = 451	*n* = 91	*n* = 4463	*n* = 3588	*n* = 875
		Median (IQR)	Median (IQR)	Median (IQR)	Median (IQR)	Median (IQR)	Median (IQR)
Age at admission (years)^ab^		82.0 (76.0–88.0)	83.0 (76.0–88.0)	81.0 (73.0–85.0)*	84.0 (78.0–89.0)	84.0 (78.0–89.0)	84.0 (78.0–89.0)
Number of comorbidities^ab^		3.0 (2.0–4.0)	3.0 (2.0–4.0)	3.0 (2.0–5.0)	2.0 (1.0–3.0)	2.0 (1.0–3.0)	2.0 (1.0–3.0)*

		*n* (%)	*n* (%)	*n* (%)	*n* (%)	*n* (%)	*n* (%)
Sex	Male	132 (24.4)	114 (25.3)	18 (19.8)	1216 (27.2)	963 (26.8)	253 (28.9)
	Female	410 (75.6)	337 (74.7)	73 (80.2)	3247 (72.8)	2625 (73.2)	622 (71.1)
Prefracture ambulation^b^	Indoor and outdoors	335 (64.1)	282 (63.2)	60 (65.9)	3159 (74.6)	2584 (72.5)	723 (83.2)*
	Indoor only	180 (34.4)	158 (35.4)	29 (31.9)	1038 (24.5)	939 (26.4)	145 (16.7)*
	No functional mobility	8 (1.5)	6 (1.3)	2 (2.2)	38 (0.9)	39 (1.1)	1 (0.1)*
Prefracture residence^b^	Nursing/residential care	132 (25.0)	119 (26.4)	16 (17.6)	757 (17.8)	703 (19.6)	87 (9.9)*
	Own home/sheltered housing	396 (75.0)	332 (73.6)	75 (82.4)	3507 (82.2)	2882 (80.4)	788 (90.1)*
Hip fracture type^b^	Intracapsular	226 (41.7)	193 (42.8)	33 (36.3)	1819 (40.8)	1485 (41.4)	334 (38.2)
	Intertrochanteric/subtrochanteric	316 (58.3)	258 (57.2)	58 (63.7)	2643 (59.2)	2103 (58.6)	540 (61.8)
Surgery within the target time^b^	Within target time	131 (25.2)	107 (24.8)	24 (27.3)	1138 (26.3)	911 (26.2)	227 (27.1)
	Not within a target time	389 (74.8)	325 (75.2)	64 (72.7)	3181 (73.7)	2569 (73.8)	612 (72.9)
First mobilisation day of/day after surgery^b^	Within the 36-h target time	408 (75.4)	328 (72.9)	80 (87.9) *	3634 (81.8)	2846 (79.7)	788 (90.4)*
	After the 36-h target time	133 (24.6)	122 (27.1)	11 (12.1) *	808 (18.2)	724 (20.3)	84 (9.6)*

*IQR* interquartile range

*p < 0.05, difference between 0–5 days frequency and 6–7 days frequency. Continuous variables compared using Mann–Whitney *U* test, categorical variables were compared using Chi-squared test

^a^Continuous variables

^b^Does not include the following missing data: age *n* = 1; prefracture ambulation *n* = 38; hip fracture type *n* = 1; surgery within target time *n* = 174; mobilisation day of/after surgery *n* = 22; prefracture residence *n* = 3; number of comorbidities *n* = 378

**Table 2:** Characteristics of 5005 patients surgically treated for non-pathological hip fracture by depression diagnosis and Duration of physiotherapy

		Depression		No depression	
		Duration		Duration	
		< 2 h	≥ 2 h	< 2 h	≥ 2 h
		*n* = 300	*n* = 228	*n* = 2275	*n* = 1992
		Median (IQR)	Median (IQR)	Median (IQR)	Median (IQR)
Age at admission (years)^ab^		83.0 (76.0–89.0)	81.0 (75.0–87.0)*	85.0 (78.0–90.0)	84.0 (78.0–89.0)*
Number of comorbidities^ab^		3.0 (2.0–4.0)	3.0 (2.0–4.0)	2.0 (1.0–3.0)	2.0 (1.0–3.0)*

		*n* (%)	*n* (%)	*n* (%)	*n* (%)
Sex	Male	74 (24.7)	56 (24.6)	620 (27.3)	545 (27.4)
	Female	226 (75.3)	172 (75.4)	1655 (72.7)	1447 (72.6)
Prefracture ambulation^b^	Indoor and outdoors	180 (60.8)	155 (68.3)	1585 (70.3)	1574 (79.5)*
	Indoor only	110 (37.2)	70 (30.8)	639 (28.3)	399 (20.1)*
	No functional mobility	6 (2.0)	2 (0.9)	30 (1.3)	8 (0.4)*
Prefracture residence^b^	Nursing/residential care	87 (29.0)	45 (19.7)*	513 (22.6)	244 (12.2)*
	Own home/sheltered housing	213 (71.0)	183 (80.3)*	1759 (77.4)	1748 (87.8)*
Hip fracture type^b^	Intracapsular	124 (41.3)	95 (41.7)	967 (42.5)	773 (38.8)*
	Intertrochanteric/subtrochanteric	176 (58.7)	133 (58.3)	1308 (57.5)	1218 (61.2)*
Surgery within the target time^b^	Within target time	69 (23.6)	58 (26.7)	626 (28.2)	463 (24.2)*
	Not within target time	223 (76.4)	159 (73.3)	1594 (71.8)	1448 (75.8)*
First mobilisation day of/day after surgery^b^	Within 36-h target time	217 (72.3)	180 (79.3)	1768 (78.3)	1707 (85.9)*
	After 36-h target time	83 (27.7)	47 (20.7)	490 (21.7)	281 (14.1)*

*IQR* interquartile range

*p < 0.05, difference between duration less than 2 h and more than 2 h. Continuous variables compared using Mann–Whitney *U* test, and categorical variables compared using Chi-squared test

^a^Continuous variables

^b^Does not include the following missing data: age *n* = 1; prefracture ambulation *n* = 38; hip fracture type *n* = 1; surgery within target time *n* = 174; mobilisation day of/after surgery *n* = 22; prefracture residence *n* = 3; number of comorbidities *n* = 378

### Discharge home

Among those with depression, 407 (75.1%) patients were admitted from home and 177 (43.5%) of these were discharged home. In those without depression, 3670 (82.3%) patients were admitted from home and 1740 (47.4%) of these were discharged home.

**Fig. 2:**
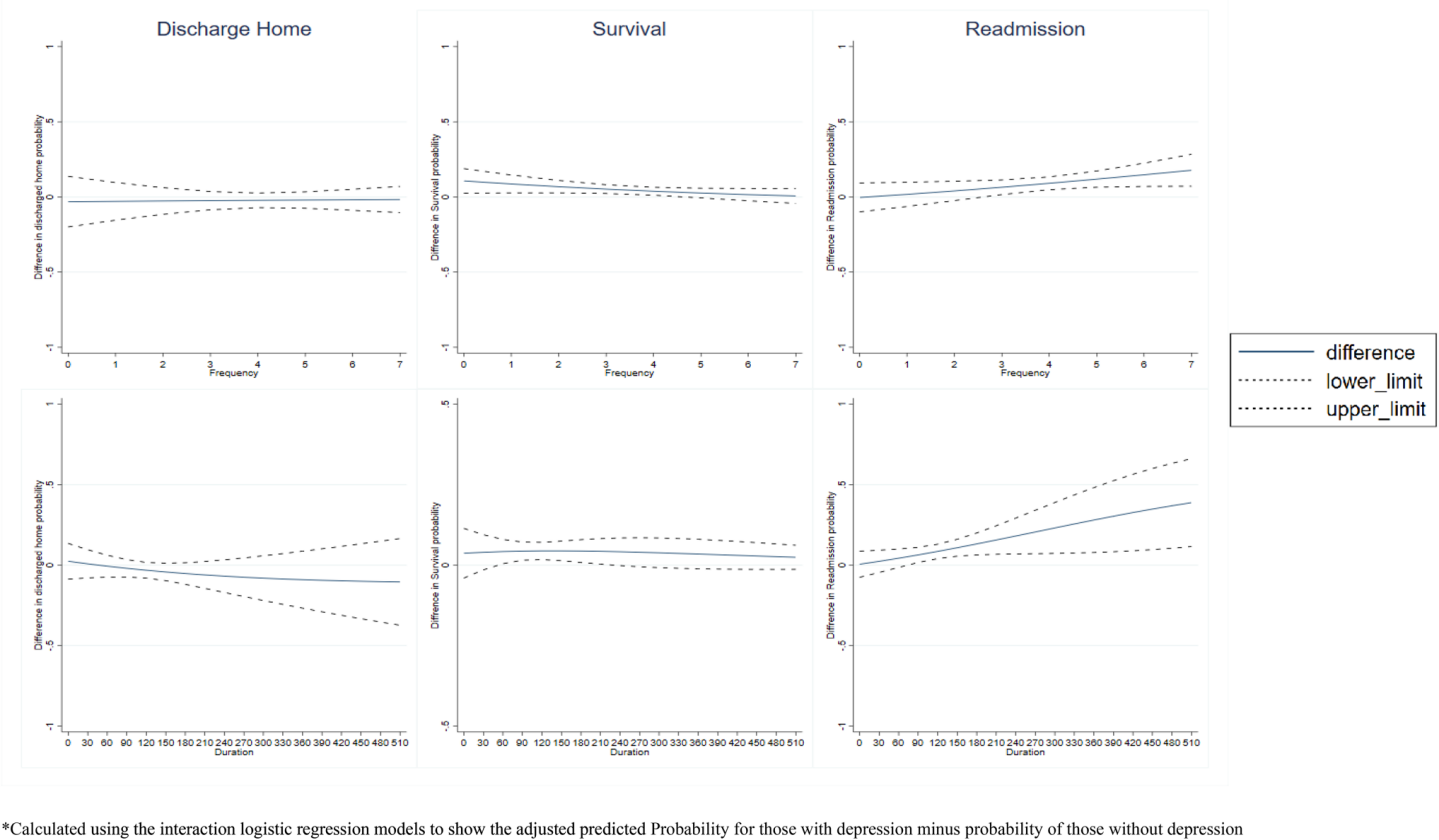
Difference in the adjusted predicted probability and their 95% CI, for those with and without a diagnosis of depression, of discharge home, survival at 30-days and avoiding readmission at 30-days by frequency and duration of physiotherapy. *Calculated using the interaction logistic regression models to show the adjusted predicted Probability for those with depression minus probability of those without depression

The average adjusted odds of discharge home for a 1-day increase in physiotherapy frequency were odds ratio (OR) 1.06 (95% CI 0.79–1.41) and 1.11 (95% CI 0.99–1.24) for those with and without depression, respectively (Table 3). There was no evidence of a formal interaction (*p* = 0.65) (Table 4, Fig. 2). The average adjusted odds of discharge home for a 30-min increase in physiotherapy duration were OR 1.05 (95% CI 0.85–1.29) and 1.16 (95% CI 1.05–1.28) for those with and without depression, respectively (Table 3). There was no evidence of a formal interaction (*p* = 0.36) (Table 4, Fig. 2).

**Table 3:** The association between duration and frequency of physiotherapy and discharge home, survival, and readmission by a diagnosis of depression

Exposure	Diagnosis of depression (*n* = 542)		No diagnosis of depression (*n* = 4463)	
	Unadjusted odds ratio (95% CI)	Adjusted odds ratio (95% CI)^a^	Unadjusted odds ratio (95% CI)	Adjusted odds ratio (95% CI)^a^
Discharge home				
Frequency				
1-day increase in physiotherapy	1.06 (0.82–1.37)	1.06 (0.79–1.41)	1.10 (0.99–1.22)	1.11 (0.99–1.24)
Duration				
30-min increase in physiotherapy	1.09 (0.89–1.34)	1.05 (0.85–1.29)	1.16 (1.06–1.27)*	1.16 (1.05–1.28)**
Survival at 30-days post-admission				
Frequency				
1-day increase in physiotherapy	1.23 (1.04–1.46)*	1.15 (0.95–1.39)	1.30 (1.23–1.38)***	1.19 (1.12–1.27)***
Duration				
30-min increase in physiotherapy	1.30 (1.10–1.54)**	1.26 (1.06–1.50)**	1.19 (1.13–1.26)***	1.11 (1.05–1.17)***
Readmission at 30-days post-discharge				
Frequency				
1-day increase in physiotherapy	1.16 (1.03–1.31)*	1.05 (1.00–1.10)*	1.16 (1.02–1.31)*	1.04 (0.99–1.09)
Duration				
30-min increase in physiotherapy	1.14 (1.04–1.25)**	1.12 (1.02–1.24)*	1.04 (1.00–1.08)*	1.03 (0.996–1.07)^b^

95% confidence intervals in brackets

*p < 0.05, **p < 0.01, ***p < 0.001

^a^With adjustment for confounders variables: age, sex, prefracture residence (not for discharge home analysis), fracture type, mobility prior to hip fracture, timing of surgery, timing of first mobilisation and a number of comorbidities. 3360 and 3444 cases with missing data of at least one of these confounder variables were excluded from the frequency and duration analyses respectively for discharge home. 820 and 936 cases with missing data of at least one of these confounder variables were excluded from frequency and duration analyses respectively for survival at 30 days. 381 and 831 cases with missing data of at least one of these confounder variables were excluded from the frequency and duration analyses respectively for readmission by 30 days

^b^Included extra decimal point to show CI

**Table 4:** The interaction models of a depression diagnosis on the association between duration and frequency of rehabilitation and discharge home, survival, and readmission

Exposure	Unadjusted odds ratio (95% CI)^a^	*p*-value	Adjusted odds ratio (95% CI)^a^	*p*-value
Discharge home				
Frequency				
1-day increase in physiotherapy	0.96 (0.73–1.27)	0.79	0.94 (0.70–1.25)	0.65
Duration				
30-min increase in physiotherapy	0.94 (0.75–1.17)	0.57	0.90 (0.72–1.13)	0.36
Survival at 30-days post-admission				
Frequency				
1-day increase in physiotherapy	0.95 (0.79–1.13)	0.55	0.91 (0.75–1.10)	0.35
Duration				
30-min increase in physiotherapy	1.09 (0.91–1.30)	0.34	1.07 (0.90–1.27)	0.45
Readmission by 30-days post-discharge				
Frequency				
1-day increase in physiotherapy	1.11 (0.97–1.26)	0.13	1.12 (0.98–1.28)	0.09
Duration				
30-min increase in physiotherapy	1.10 (0.99–1.21)	0.07	1.10 (0.99–1.21)	0.07

^a^With adjustment set

### 30-day survival

In those with depression, 472 (87.9%) patients were alive by 30 days post-admission, while for those without depression, 3764 (86.6%) patients were alive by 30 days post-admission.

The average adjusted odds of survival for a 1-day increase in physiotherapy frequency were OR 1.15 (95% CI 0.95–1.39) and 1.19 (95% CI 1.12–1.27) for those with and without depression, respectively (Table 3). There was no evidence of a formal interaction (*p* = 0.35) (Table 4, Fig. 2). The average adjusted odds of survival for a 30-min increase in physiotherapy duration were OR 1.26 (95% CI 1.06–1.50) and 1.11 (95% CI 1.05–1.17) for those with and without depression, respectively (Table 3). There was no evidence of a formal interaction (*p* = 0.45) (Table 4, Fig. 2).

### Readmission at 30-days

In those with depression, 365 (67.3%) patients were readmitted within 30 days of discharge while 3339 (74.8%) patients without depression were readmitted within 30 days of discharge.

The average adjusted odds of readmission for a 1-day increase in physiotherapy frequency were OR 1.16 (95% CI 1.02–1.31) and 1.04 (95% CI 0.99–1.09) for those with and without depression, respectively (Table 3). There was no evidence of a formal interaction (*p* = 0.09) (Table 4, Fig. 2). The average adjusted odds of readmission for a 30-min increase in physiotherapy duration were OR 1.12 (95% CI 1.02–1.24) and 1.03 (95% CI 0.996–1.07) for those with and without depression, respectively (Table 3). There was no evidence of a formal interaction (*p* = 0.09) (Table 4, Fig. 2).

### Sensitivity analyses

Sensitivity analyses where outcome and exposure data were imputed for patients with missing data are presented in Online Resource 1. Baseline characteristics for the overall, complete case and excluded samples are presented in Online Resource 1. Similar findings were found for survival for the complete case and imputed analyses. Significant effects were seen for the unadjusted frequency-discharge and adjusted duration-readmission models in those without depression for the imputed analyses but not for the complete case (Online Resource 1). All other models were comparable for the complete case and imputed analyses (Online Resource 1). Additionally, we conducted sensitivity checks for the discharge home models, including those who died in the hospital to the group of “no discharge home”. It is likely that if these individuals had been alive, they would have had some level of dependency such as requiring discharge to a nursing/residential care home. This however did not alter the results. A further sensitivity check was performed as a small number of recorded durations of physiotherapy appeared implausibly high. However, results were similar with no changes in the magnitude or significance of any effect after excluding 28 participants with durations above 320 min.

## Discussion

### Summary of results

The results of this study suggest comparable but some differences in the association between physiotherapy frequency and duration and outcomes between groups with and without depression. However, there was no significant formal interaction of a depression diagnosis in the association between physiotherapy frequency and duration and outcomes.

### Our contribution to the literature

A recent Cochrane review concluded there may be a beneficial effect of in-hospital rehabilitation after hip fracture surgery in reducing the likelihood of outcomes such as death, admission to institutional care and readmission [33]. Handoll et al. [33] found low level evidence for an association between rehabilitation and reduced chance of admission to residential care for all patients and lower mortality rate among those with poor mobility, but no association with readmission. We found similar results in the present study for patients without depression but not for patients with depression. This supports the hypothesis that patients with depression are a separate heterogeneous group within the hip fracture population.

The research has largely focused on the barriers to exercise participation in patients with depression. Namely, the belief that depression interferes with exercise participation due to its common characteristics of a reduction in interest, motivation, energy, confidence, and fatigue [34]. Yet, the evidence on how depression affects an individual’s ability to access physiotherapy is limited. Here, depression diagnosis did not appear to influence the amount of frequency and duration of physiotherapy received by hip fracture patients overall. This suggests a diagnosis of depression does not impact an individual’s ability to access physiotherapy in the first postoperative week after their hip fracture. However, patients included in this study were those with a formal diagnosis of depression and not those with depressive symptoms more broadly. Therefore, whether there is equity in physiotherapy frequency and duration amongst those along the spectrum of depression and depressive symptoms remains to be explored.

For patients with depression, greater physiotherapy frequency and duration were associated with higher odds of readmission at 30 days post-discharge. An association was not evident in patients without depression after full adjustment. The interaction models were also suggestive of a stronger effect of greater physiotherapy frequency and duration being associated with a higher likelihood of readmission for patients with depression compared to those without depression. Previous literature suggests those who are depressed are more likely to be re-admitted at 30 days following orthopaedic surgery [11, 12]. This may be because patients with depression are more likely to be frailer, have multi-morbidities and have lower functional independence compared to those without depression. Meaning they have a higher chance of readmission to the hospital after a hip fracture surgery [35]. The baseline characteristics of patients with and without depression in this study also support this. Those with depression tended to have more comorbidities, be residents of nursing or residential homes and were more likely to have no functional mobility than those without depression.

The results of readmission stratified and interaction models may therefore suggest a confounding by indication role of depression [36]. Confounding by indication or severity is the phenomenon that occurs when the patient’s clinical indication is associated with both the treatment approach and the outcomes. This may present as patients who are more severely ill receiving more or more intense intervention which seemingly results in poorer outcomes compared to those receiving less or less intense intervention [36]. In the current study, it may be that patients with depression are frailer. Therefore, they require more physiotherapy. However, due to their more severe level of morbidity this results in more chances of readmission than those without depression. This highlights the complexities and multifaceted nature of the relationship between depression, physiotherapy frequency and duration and patient outcomes. To further understand the effect of physiotherapy on readmission in those with depression it is important to ascertain if receiving less physiotherapy for these patients, results in even more readmissions than those receiving more physiotherapy.

The interaction models for the survival outcome suggest there is no formal interaction between greater physiotherapy frequency and duration and a diagnosis of depression in their association with a higher likelihood of survival. The association between the duration of physiotherapy and survival at 30 days post admission was not influenced by depression diagnosis in the stratified logistic models. The association between the frequency of physiotherapy and survival also showed similar effect estimates in the stratified models. However, the lack of significance observed in patients with depression may be due to a lack of power. This may also be related to the inclusion of patients with a formal depression diagnosis only in the current study. A previous study found evidence that for patients with depression, exercise training may improve survival in those with coronary heart disease [37]. Interestingly, the authors found this benefit was achieved by reducing patients’ depressive symptoms. Amongst patients with depression and heart disease at the start of the study, those who remained depressed by the study end had a fourfold difference in their mortality rate compared to those who were no longer depressed [37].

It could be that the associations between physiotherapy frequency and duration and survival in those with depression are more apparent when the population includes those along the spectrum of depression symptoms versus only those with a diagnosis of depression. To confirm the association between physiotherapy frequency and duration and survival in patients with depression and hip fracture, investigations are warranted on those with varying levels of depression or depressive symptoms at study commencement to explore differences in survival rates between those with and without depression at study end.

A diagnosis of depression did not impact the lack of association between the frequency of physiotherapy and discharge home in the stratified and interaction models. In patients without depression, a longer duration of physiotherapy was associated with higher odds of discharge home but not for patients with depression. In the interaction duration-discharge home model, the results suggest no formal interaction between greater physiotherapy duration and a diagnosis of depression in their association with a higher likelihood of discharge home. The lack of association between duration of physiotherapy and discharge home in patients with depression in the stratified model is in keeping with previous evidence which suggested a higher likelihood of being discharged to a nursing home in older hip fracture patients with depression compared to those without depression [38]. This has been hypothesised to be due to the relationship between depression and low social support. Often those with depression have the lower social capital to employ to avoid admission to residential/nursing care compared with their non-depressed counterparts [38]. The current study suggests additional physiotherapy frequency and duration during the first postoperative week does not mitigate a lack of social capital in those with depression. The effectiveness of interventions targeted at improving social capital remains unclear [39]. Therefore, whether there is a benefit for ongoing physiotherapy across the continuum of care is still unknown.

From the regression models analysed it appears the model’s involving the duration of physiotherapy showed stronger effects than physiotherapy frequency models. These results may suggest longer physiotherapy sessions for patients may equate to better outcomes than receiving more days of physiotherapy for both patients with and without depression. This contradicts the hip fracture management evidence that suggests daily rehabilitation is better accepted than longer, less frequent sessions [40]. Although this guidance did not consider the impact of physiotherapy on patient outcomes and only assessed tolerability [40]. It is possible that these patients, who often have complex needs and histories, may require more time to build rapport and work through all the necessary treatment components within a given session. Therefore, supporting the need for longer and less frequent sessions.

A further hypothesis is that shorter, regular physiotherapy sessions become more functionally focused with less psychological and holistic support. Given the traumatic nature of a hip fracture event it is a common occurrence for patients with hip fracture to think about the end of life for the first time [41]. Therefore, sessions encompassing these psychological aspects may result in better outcomes. However, these hypotheses must be confirmed through investigations on varying levels of physiotherapy frequencies and durations and their respective impact on patient outcomes in patients post-hip fracture surgery.

### Strengths and limitations

The strengths of this study include the large nationally representative sample size taken from a national audit and the availability of data for several confounding variables. There are limitations to this study. Disproportional sample sizes of those with and without a diagnosis of depression may explain why the formal interaction models showed a non-significant association between physiotherapy frequency and duration and outcomes, even when differences were evident in the separate models between patients with and without depression. This was despite our power calculations indicating adequately powered models. Next, medication use, and detail was not part of the data collected. Therefore, the confounding nature of antidepressants use in the investigated associations is unknown. Patients with depression were identified by the presence/absence of an ICD-10 diagnosis code for depression during the hip fracture hospitalisation or in the year prior to this hospitalisation. Additional data on the diagnosis time, severity, associated treatment, or whether the patients were symptomatic or not were not available. These factors may influence the association between physiotherapy and outcomes given the relationship between a key feature of depression, lack of engagement/interest, and poor outcomes [34] and the side effects of antidepressant use and physical symptoms such as nausea, unsteadiness and dizziness which may impact one’s ability to engage with physiotherapy [42]. Next, despite including several confounder variables in our adjusted models there is still the potential for confounding from other variables such as deprivation and Hospital Frailty risk score not included in the analysis. The duration variable taken from the PHFSA may have included data entry errors as a patient receiving 511 min of physiotherapy in one week may not be likely. However, upon limiting the maximum duration to 320 min and re-running the analysis, there were minimal differences. Last, there was missing data for the exposure variable of physiotherapy duration, the outcomes, and various confounding variables. However, we assessed the impact of these missing data on our results through multiple imputation by chained equations and found most results comparable between the complete case and the imputed analyses. Missing data for the outcome of discharge home was likely not to be missing at random as some sites had greater access to inpatient rehabilitation than others. This may mean the results of this study may not be generalisable to patients who are not able to access equivalent levels of inpatient rehabilitation. However, we adopted completed cases only for our main analysis and upon comparing baseline characteristics for the overall and complete case samples, we found comparable results. Given many of these limitations are due to the retrospective nature of the study, a prospective study would improve the strength of the results.

## Conclusions

The results of this study indicate physiotherapy frequency and duration are similar between those with and without a diagnosis of depression. There was no significant formal interaction effect of the presence of depression in the association between physiotherapy frequency and duration and outcomes, but the test for the readmission model was close. Although, readmission was associated with physiotherapy frequency and duration in those with depression but not those without depression and results suggest duration of physiotherapy may have a greater impact on outcomes compared to the frequency of physiotherapy. Considering these results, prospective studies should be conducted to confirm/refute the signal of an association between the longer duration of postoperative physiotherapy in patients with hip fracture and depression and readmission observed here. Additionally, future research should further assess varying levels of frequency and duration on the associations with outcomes and the impact of a formal depression diagnosis as well as various severities in depressive symptoms on this relationship.

### Supplementary Information

Below is the link to the electronic supplementary material.

Supplementary file1 (DOCX 73 kb)

## Data Availability

The data used in this study is available from NHS Digital, NHS Wales Informatics Service, the Royal College of Physician’s Falls and Fragility Fracture Audit programme and Healthcare Quality Improvement Partnership. However, restrictions apply to the availability of these data, which were used under license for the current study, and so are not publicly available.
