# Supplementary material for: The role of depression in the association between physiotherapy frequency and duration and outcomes after hip fracture surgery: secondary analysis of the physiotherapy hip fracture sprint audit
Source: Eur Geriatr Med. 2023 Jun 17;14(5):999–1010. doi: 10.1007/s41999-023-00808-8 (PMC10587201; doi:10.1007/s41999-023-00808-8)
Supplement: Supplementary file 1 — Supplementary file1 (DOCX 73 kb) [file 41999_2023_808_MOESM1_ESM.docx]

|  | Diagnosis of depression (n=542) | | No diagnosis of depression (n=4,463) | |
| --- | --- | --- | --- | --- |
| Exposure | Unadjusted Model  n (%) | Adjusted Model n (%) | Unadjusted Model n (%) | Adjusted Model n (%) |
| Discharge home | | | | |
| Frequency | | | | |
| 1-day increase in physiotherapy | 210 (38.75) | 202 (37.27) | 1950 (43.69) | 1859 (41.65) |
| Duration | | | | |
| 30-minute increase in physiotherapy | 206 (38.01) | 191 (35.24) | 1864 (41.77) | 1748 (39.17) |
| Survival at 30-days post-admission | | | | |
| Frequency | | | | |
| 1-day increase in physiotherapy | 537 (99.08) | 502 (92.62) | 4345 (97.36) | 4164 (93.30) |
| Duration | | | | |
| 30-minute increase in physiotherapy | 523 (96.49) | 491 (90.59) | 4158 (93.17) | 3956 (88.63) |
| Readmission by 30-days post-discharge | | | | |
| Frequency | | | | |
| 1-day increase in physiotherapy | 542 (100) | 542 (100) | 4463 (100) | 4460 (99.93) |
| Duration | | | | |
| 30-minute increase in physiotherapy | 528 (97.42) | 495 (91.33) | 4267 (95.61) | 4057 (90.90) |

Table S1: The number of patients included in each stratified model

Note: The percentages are the patients in the depression and no depression groups respectively included in the regression models

Table S2: The number of patients included in each Interaction Model. Total = 5,005

| Exposure | Unadjusted Model  n (%) | Adjusted Model  n (%) |
| --- | --- | --- |
| Discharge home | | |
| Frequency | | |
| 1-day increase in physiotherapy | 2160 (43.16) | 2071 (41.38) |
| Duration | | |
| 30-minute increase in physiotherapy | 2070 (41.36) | 1984 (39.64) |
| Survival at 30-days post-admission | | |
| Frequency | | |
| 1-day increase in physiotherapy | 4882 (97.54) | 4674 (93.39) |
| Duration | | |
| 30-minute increase in physiotherapy | 4681 (93.53) | 4483 (89.57) |
| Readmission by 30-days post-discharge | | |
| Frequency | | |
| 1-day increase in physiotherapy | 5005 (100) | 5002 (99.94) |
| Duration | | |
| 30-minute increase in physiotherapy | 4795 (95.80) | 4590 (91.71) |

| **Discharge home Models** | **Complete Case** | | | | **Excluded** | | | |
| --- | --- | --- | --- | --- | --- | --- | --- | --- |
|  | **Depression** | | **No Depression** | | **Depression** | | **No Depression** | |
| **Physiotherapy Duration** | **< 2 hours** | **≥ 2 hours** | **< 2 hours** | **≥ 2 hours** | **< 2 hours** | **≥ 2 hours** | **< 2 hours** | **≥ 2 hours** |
|  | **n = 111** | **n = 89** | **n = 839** | **n = 935** | **n = 189** | **n = 139** | **n = 1,436** | **n = 1,057** |
|  | **median**  **[IQR]** | **median [IQR]** | **median**  **[IQR]** | **median**  **[IQR]** | **median**  **[IQR]** | **median [IQR]** | **median**  **[IQR]** | **median**  **[IQR]** |
| Age at admission (years)* | 79.0 (72.0-86.0) | 79.0 (71.0-83.0) | 81.0 (73.0-87.0) | 82.0 (75.0-87.0) * | 85.0 (79.0-91.0) | 83.0 (78.0-88.0) | 86.0 (81.0-91.0) | 85.0 (80.0-90.0) * |
| Number of comorbidities* | 3.0 (2.0-4.0) | 2.0 (2.0-4.0) | 1.0 (1.0-3.0) | 1.0 (1.0-2.0) | 3.0 (2.0-5.0) | 4.0 (2.0-5.0) | 2.0 (1.0-3.0) | 2.0 (1.0-3.0) * |
|  | **n (%)** | **n (%)** | **n (%)** | **n (%)** | **n (%)** | **n (%)** | **n (%)** | **n (%)** |
| Sex |  |  |  |  |  |  |  |  |
| Male | 30 (27.0) | 22 (24.7) | 229 (27.3) | 273 (29.2) | 44 (23.3) | 34 (24.5) | 391 (27.2) | 272 (25.7) |
| Female | 81 (73.0) | 67 (75.3) | 610 (72.7) | 662 (70.8) | 145 (76.7) | 105 (75.5) | 1,045 (72.8) | 785 (74.3) |
| Prefracture ambulation |  |  |  |  |  |  |  |  |
| Indoor & outdoors | 75 (67.6) | 73 (82.0) | 700 (83.4) | 820 (87.7) * | 105 (56.8) | 82 (59.4) | 885 (62.5) | 754 (72.1) * |
| Indoor only | 33 (29.7) | 15 (16.9) | 139 (16.6) | 115 (12.3) * | 77 (41.6) | 55 (39.9) | 500 (35.3) | 284 (27.2) * |
| No functional mobility | 3 (2.7) | 1 (1.1) | - | - | 3 (1.6) | 1 (0.7) | 30 (2.1) | 8 (0.8) * |
| Prefracture residence |  |  |  |  |  |  |  |  |
| Nursing/Residential Care | - | - | - | - | 87 (46.0) | 45 (32.4) * | 513 (35.8) | 244 (23.1) * |
| Own home/sheltered housing | 111 (100.0) | 89 (100.0) | 839 (100.0) | 935 (100.0) | 102 (54.0) | 94 (67.6) * | 920 (64.2) | 813 (76.9) * |
| Hip fracture type |  |  |  |  |  |  |  |  |
| Intracapsular | 42 (37.8) | 34 (38.2) | 307 (36.6) | 310 (33.2) | 82 (43.4) | 61 (43.9) | 660 (46.0) | 463 (43.8) |
| Intertrochanteric/ Subtrochanteric | 69 (62.2) | 55 (61.8) | 532 (63.4) | 625 (66.8) | 107 (56.6) | 78 (56.1) | 776 (54.0) | 593 (56.2) |
| Surgery within the target time |  |  |  |  |  |  |  |  |
| Within target time | 30 (27.0) | 23 (25.8) | 250 (29.8) | 201 (21.5) * | 39 (21.5) | 35 (27.3) | 376 (27.2) | 262 (26.8) |
| Not within target time | 81 (73.0) | 66 (74.2) | 589 (70.2) | 734 (78.5) * | 142 (78.5) | 93 (72.7) | 1,005 (72.8) | 714 (73.2) |
| First mobilisation day of/day after surgery |  |  |  |  |  |  |  |  |
| Within 36-hour target time | 84 (75.7) | 78 (87.6) * | 709 (84.5) | 838 (89.6) * | 133 (70.4) | 102 (73.9) | 1,059 (74.6) | 869 (82.5) * |
| After 36-hour target time | 27 (24.3) | 11 (12.4) * | 130 (15.5) | 97 (10.4) * | 56 (29.6) | 36 (26.1) | 360 (25.4) | 184 (17.5) * |

Table S3. Characteristics of 4,795 patients surgically treated for non-pathological hip fracture by depression diagnosis and physiotherapy duration and inclusion/exclusion in complete case analysis (discharge home).

*p<0.05

Table S4. Characteristics of 4,795 patients surgically treated for non-pathological hip fracture by depression diagnosis and physiotherapy duration and inclusion/exclusion in complete case analysis (survival at 30-days).

| **Survival** **Models** | **Complete Case** | | | | **Excluded** | | | |
| --- | --- | --- | --- | --- | --- | --- | --- | --- |
|  | **Depression** | | **No Depression** | | **Depression** | | **No Depression** | |
| **Physiotherapy Duration** | **< 2 hours** | **≥ 2 hours** | **< 2 hours** | **≥ 2 hours** | **< 2 hours** | **≥ 2 hours** | **< 2 hours** | **≥ 2 hours** |
|  | **n = 280** | **n = 211** | **n = 2,139** | **n = 1,845** | **n = 20** | **n = 17** | **n = 136** | **n = 147** |
|  | **median**  **[IQR]** | **median [IQR]** | **median**  **[IQR]** | **median**  **[IQR]** | **median**  **[IQR]** | **median [IQR]** | **median**  **[IQR]** | **median**  **[IQR]** |
| Age at admission (years)* | 83.0 (76.0-89.0) | 82.0 (76.0-87.0) | 85.0 (78.0-90.0) | 84.0 (78.0-89.0) * | 85.5 (78.5-90.0) | 74.0 (71.0-81.0) * | 83.0 (74.5-88.0) | 82.0 (76.0-87.0) |
| Number of comorbidities* | 3.0 (2.0-4.0) | 3.0 (2.0-4.0) | 2.0 (1.0-3.0) | 2.0 (1.0-3.0) * | 4.0 (3.5-5.0) | 2.0 (2.0-4.0) * | 1.0 (1.0-2.0) | 1.0 (1.0-2.0) |
|  | **n (%)** | **n (%)** | **n (%)** | **n (%)** | **n (%)** | **n (%)** | **n (%)** | **n (%)** |
| Sex |  |  |  |  |  |  |  |  |
| Male | 68 (24.3) | 52 (24.6) | 584 (27.3) | 510 (27.6) | 6 (30.0) | 4 (23.5) | 36 (26.5) | 35 (23.8) |
| Female | 212 (75.7) | 159 (75.4) | 1,555 (72.7) | 1,335 (72.4) | 14 (70.0) | 13 (76.5) | 100 (73.5) | 112 (76.2) |
| Prefracture ambulation |  |  |  |  |  |  |  |  |
| Indoor & outdoors | 171 (61.1) | 144 (68.2) | 1,486 (69.5) | 1,459 (79.1) | 9 (56.3) | 11 (68.8) | 99 (86.1) | 115 (84.6) |
| Indoor only | 109 (38.9) | 67 (31.8) | 624 (29.2) | 378 (20.5) | 1 (6.3) | 3 (18.8) | 15 (13.0) | 21 (15.4) |
| No functional mobility | - | - | 29 (1.4) | 8 (0.4) | 6 (37.5) | 2 (12.5) | 1 (0.9) | 0 (0.0) |
| Prefracture residence |  |  |  |  |  |  |  |  |
| Nursing/Residential Care | 78 (27.9) | 40 (19.0) * | 493 (23.0) | 235 (12.7) * | 9 (45.0) | 5 (29.4) | 20 (15.0) | 9 (6.1) * |
| Own home/sheltered housing | 202 (72.1) | 171 (81.0) * | 1,646 (77.0) | 1,610 (87.3) * | 11 (55.0) | 12 (70.6) | 113 (85.0) | 138 (93.9) * |
| Hip fracture type |  |  |  |  |  |  |  |  |
| Intracapsular | 116 (41.4) | 90 (42.7) | 913 (42.7) | 731 (39.6) * | 8 (40.0) | 5 (29.4) | 54 (39.7) | 42 (28.8) * |
| Intertrochanteric/ Subtrochanteric | 164 (58.6) | 121 (57.3) | 1,226 (57.3) | 1,114 (60.4) * | 12 (60.0) | 12 (70.6) | 82 (60.3) | 104 (71.2) * |
| Surgery within the target time |  |  |  |  |  |  |  |  |
| Within target time | 64 (22.9) | 57 (27.0) | 600 (28.1) | 448 (24.3) * | 5 (41.7) | 1 (16.7) | 26 (32.1) | 15 (22.7) |
| Not within target time | 216 (77.1) | 154 (73.0) | 1,539 (71.9) | 1,397 (75.7) * | 7 (58.3) | 5 (83.3) | 55 (67.9) | 51 (77.3) |
| First mobilisation day of/day after surgery |  |  |  |  |  |  |  |  |
| Within 36-hour target time | 208 (74.3) | 166 (78.7) | 1,674 (78.3) | 1,583 (85.8) * | 9 (45.0) | 14 (87.5) * | 94 (79.0) | 124 (86.7) |
| After 36-hour target time | 72 (25.7) | 45 (21.3) | 465 (21.7) | 262 (14.2) * | 11 (55.0) | 2 (12.5) * | 25 (21.0) | 19 (13.3) |

*p<0.05

Table S5. Characteristics of 4,795 patients surgically treated for non-pathological hip fracture by depression diagnosis and physiotherapy duration and inclusion/exclusion in complete case analysis (Readmission at 30-days).

| **Readmission Models** | **Complete Case** | | | | **Excluded** | | | |
| --- | --- | --- | --- | --- | --- | --- | --- | --- |
|  | **Depression** | | **No Depression** | | **Depression** | | **No Depression** | |
| **Physiotherapy Duration** | **< 2 hours** | **≥ 2 hours** | **< 2 hours** | **≥ 2 hours** | **< 2 hours** | **≥ 2 hours** | **< 2 hours** | **≥ 2 hours** |
|  | **n = 288** | **n = 215** | **n = 2,190** | **n = 1,897** | **n = 12** | **n = 13** | **n = 85** | **n = 95** |
|  | **median**  **[IQR]** | **median [IQR]** | **median**  **[IQR]** | **median**  **[IQR]** | **median**  **[IQR]** | **median [IQR]** | **median**  **[IQR]** | **median**  **[IQR]** |
| Age at admission (years)* | 83.0 (76.0-89.0) | 82.0 (76.0-87.0) | 85.0 (78.0-90.0) | 84.0 (78.0-89.0) | 87.0 (82.0-91.5) | 75.0 (71.0-84.0) * | 83.0 (72.0-88.0) | 81.0 (74.0-87.0) |
| Number of comorbidities* | 3.0 (2.0-4.0) | 3.0 (2.0-4.0) | 2.0 (1.0-3.0) | 2.0 (1.0-3.0) * | 4.0 (3.0-5.0) | 3.0 (2.0-4.0) | 1.0 (1.0-3.0) | 1.0 (1.0-2.0) |
|  | **n (%)** | **n (%)** | **n (%)** | **n (%)** | **n (%)** | **n (%)** | **n (%)** | **n (%)** |
| Sex |  |  |  |  |  |  |  |  |
| Male | 71 (24.7) | 53 (24.7) | 596 (27.2) | 527 (27.8) | 3 (25.0) | 3 (23.1) | 24 (28.2) | 18 (18.9) |
| Female | 217 (75.3) | 162 (75.3) | 1,594 (72.8) | 1,370 (72.2) | 9 (75.0) | 10 (76.9) | 61 (71.8) | 77 (81.1) |
| Prefracture ambulation |  |  |  |  |  |  |  |  |
| Indoor & outdoors | 173 (60.1) | 146 (67.9) | 1,530 (69.9) | 1,503 (79.2) * | 7 (87.5) | 9 (75.0) | 55 (85.9) | 71 (84.5) |
| Indoor only | 109 (37.8) | 67 (31.2) | 631 (28.8) | 386 (20.3) * | 1 (12.5) | 3 (25.0) | 8 (12.5) | 13 (15.5) |
| No functional mobility | 6 (2.1) | 2 (0.9) | 29 (1.3) | 8 (0.4) * | - | - | 1 (1.6) | 0 (0.0) |
| Prefracture residence |  |  |  |  |  |  |  |  |
| Nursing/Residential Care | 80 (27.8) | 41 (19.1) * | 499 (22.8) | 236 (12.4) * | 7 (58.3) | 4 (30.8) | 14 (17.1) | 8 (8.4) |
| Own home/sheltered housing | 208 (72.2) | 174 (80.9) * | 1,691 (77.2) | 1,661 (87.6) * | 5 (41.7) | 9 (69.2) | 68 (82.9) | 87 (91.6) |
| Hip fracture type |  |  |  |  |  |  |  |  |
| Intracapsular | 120 (41.7) | 93 (43.3) | 935 (42.7) | 746 (39.3) * | 4 (33.3) | 2 (15.4) | 32 (37.6) | 27 (28.7) |
| Intertrochanteric/ Subtrochanteric | 168 (58.3) | 122 (56.7) | 1,255 (57.3) | 1,151 (60.7) * | 8 (66.7) | 11 (84.6) | 53 (62.4) | 67 (71.3) |
| Surgery within the target time |  |  |  |  |  |  |  |  |
| Within target time | 67 (23.3) | 58 (27.0) | 614 (28.0) | 458 (24.1) * | 2 (50.0) | 0 (0.0) | 12 (40.0) | 5 (35.7) |
| Not within target time | 221 (76.7) | 157 (73.0) | 1,576 (72.0) | 1,439 (75.9) * | 2 (50.0) | 2 (100.0) | 18 (60.0) | 9 (64.3) |
| First mobilisation day of/day after surgery |  |  |  |  |  |  |  |  |
| Within 36-hour target time | 212 (73.6) | 169 (78.6) | 1,717 (78.4) | 1,630 (85.9) * | 5 (41.7) | 11 (91.7) * | 51 (75.0) | 77 (84.6) |
| After 36-hour target time | 76 (26.4) | 46 (21.4) | 473 (21.6) | 267 (14.1) * | 7 (58.3) | 1 (8.3) * | 17 (25.0) | 14 (15.4) |

*p<0.05

Table S6. Characteristics of 5,005 patients surgically treated for non-pathological hip fracture by depression diagnosis and physiotherapy frequency and inclusion/exclusion in complete case analysis (discharge home)

| **Discharge home Models** | **Complete Case** | | | | **Excluded** | | | |
| --- | --- | --- | --- | --- | --- | --- | --- | --- |
|  | **Depression** | | **No Depression** | | **Depression** | | **No Depression** | |
| **Physiotherapy Frequency** | **0-5 days** | **6-7 days** | **0-5 days** | **6-7 days** | **0-5 days** | **6-7 days** | **0-5 days** | **6-7 days** |
|  | **n = 157** | **n = 45** | **n = 1,399** | **n = 460** | **n = 294** | **n = 46** | **n = 2,189** | **n = 415** |
|  | **median**  **[IQR]** | **median [IQR]** | **median**  **[IQR]** | **median**  **[IQR]** | **median**  **[IQR]** | **median [IQR]** | **median**  **[IQR]** | **median**  **[IQR]** |
| Age at admission (years)* | 79.0 (72.0-84.0) | 81.0 (74.0-86.0) | 81.0 (74.0-87.0) | 82.0 (76.0-87.0) * | 84.5 (79.0-90.0) | 80.0 (73.0-85.0) * | 86.0 (81.0-91.0) | 86.0 (81.0-90.0) |
| Number of comorbidities* | 3.0 (2.0-4.0) | 2.0 (2.0-4.0) | 1.0 (1.0-3.0) | 1.0 (1.0-3.0) | 3.0 (2.0-5.0) | 4.0 (3.0-5.0) | 2.0 (1.0-3.0) | 2.0 (1.0-3.0) |
|  | **n (%)** | **n (%)** | **n (%)** | **n (%)** | **n (%)** | **n (%)** | **n (%)** | **n (%)** |
| Sex |  |  |  |  |  |  |  |  |
| Male | 43 (27.4) | 10 (22.2) | 373 (26.7) | 150 (32.6) * | 71 (24.1) | 8 (17.4) | 590 (27.0) | 103 (24.8) |
| Female | 114 (72.6) | 35 (77.8) | 1,026 (73.3) | 310 (67.4) * | 223 (75.9) | 38 (82.6) | 1,599 (73.0) | 312 (75.2) |
| Prefracture ambulation |  |  |  |  |  |  |  |  |
| Indoor & outdoors | 116 (73.9) | 33 (73.3) | 1,179 (84.3) | 414 (90.0) * | 166 (57.4) | 27 (58.7) | 1,405 (65.0) | 309 (75.6) * |
| Indoor only | 39 (24.8) | 10 (22.2) | 220 (15.7) | 46 (10.0) * | 119 (41.2) | 19 (41.3) | 719 (33.2) | 99 (24.2) * |
| No functional mobility | 2 (1.3) | 2 (4.4) | - | - | 4 (1.4) | 0 (0.0) | 39 (1.8) | 1 (0.2) * |
| Prefracture residence |  |  |  |  |  |  |  |  |
| Nursing/Residential Care | - | - | - | - | 119 (40.5) | 16 (34.8) | 703 (32.2) | 87 (21.0) * |
| Own home/sheltered housing | 157 (100.0) | 45 (100.0) | 1,399 (100.0) | 460 (100.0) | 175 (59.5) | 30 (65.2) | 1,483 (67.8) | 328 (79.0) * |
| Hip fracture type |  |  |  |  |  |  |  |  |
| Intracapsular | 62 (39.5) | 15 (33.3) | 494 (35.3) | 154 (33.5) | 131 (44.6) | 18 (39.1) | 991 (45.3) | 180 (43.5) |
| Intertrochanteric/ Subtrochanteric | 95 (60.5) | 30 (66.7) | 905 (64.7) | 306 (66.5) | 163 (55.4) | 28 (60.9) | 1,198 (54.7) | 234 (56.5) |
| Surgery within the target time |  |  |  |  |  |  |  |  |
| Within target time | 41 (26.1) | 12 (26.7) | 357 (25.5) | 111 (24.1) | 66 (24.0) | 12 (27.9) | 554 (26.6) | 116 (30.6) |
| Not within target time | 116 (73.9) | 33 (73.3) | 1,042 (74.5) | 349 (75.9) | 209 (76.0) | 31 (72.1) | 1,527 (73.4) | 263 (69.4) |
| First mobilisation day of/day after surgery |  |  |  |  |  |  |  |  |
| Within 36-hour target time | 121 (77.1) | 43 (95.6) * | 1,203 (86.0) | 420 (91.3) * | 207 (70.6) | 37 (80.4) | 1,643 (75.7) | 368 (89.3) * |
| After 36-hour target time | 36 (22.9) | 2 (4.4) * | 196 (14.0) | 40 (8.7) * | 86 (29.4) | 9 (19.6) | 528 (24.3) | 44 (10.7) * |

*p<0.05

Table S7. Characteristics of 5,005 patients surgically treated for non-pathological hip fracture by depression diagnosis and physiotherapy frequency and inclusion/exclusion in complete case analysis (survival at 30-days)

| **Survival** **Models** | **Complete Case** | | | | **Excluded** | | | |
| --- | --- | --- | --- | --- | --- | --- | --- | --- |
|  | **Depression** | | **No Depression** | | **Depression** | | **No Depression** | |
| **Physiotherapy Frequency** | **0-5 days** | **6-7 days** | **0-5 days** | **6-7 days** | **0-5 days** | **6-7 days** | **0-5 days** | **6-7 days** |
|  | **n = 416** | **n = 86** | **n = 3,357** | **n = 807** | **n = 35** | **n = 5** | **n = 231** | **n = 68** |
|  | **median**  **[IQR]** | **median [IQR]** | **median**  **[IQR]** | **median**  **[IQR]** | **median**  **[IQR]** | **median [IQR]** | **median**  **[IQR]** | **median**  **[IQR]** |
| Age at admission (years)* | 83.0 (76.0-88.0) | 81.0 (73.0-85.0) * | 84.0 (78.0-89.0) | 84.0 (78.0-89.0) | 81.0 (75.0-88.0) | 74.0 (73.0-79.0) | 82.0 (74.0-88.0) | 83.5 (77.5-86.5) |
| Number of comorbidities* | 3.0 (2.0-4.0) | 3.0 (2.0-5.0) | 2.0 (1.0-3.0) | 2.0 (1.0-3.0) * | 4.0 (2.0-5.0) | 4.0 (2.0-4.0) | 1.0 (1.0-2.0) | 1.5 (1.0-3.0) |
|  | **n (%)** | **n (%)** | **n (%)** | **n (%)** | **n (%)** | **n (%)** | **n (%)** | **n (%)** |
| Sex |  |  |  |  |  |  |  |  |
| Male | 105 (25.2) | 16 (18.6) | 907 (27.0) | 234 (29.0) | 9 (25.7) | 2 (40.0) | 56 (24.2) | 19 (27.9) |
| Female | 311 (74.8) | 70 (81.4) | 2,450 (73.0) | 573 (71.0) | 26 (74.3) | 3 (60.0) | 175 (75.8) | 49 (72.1) |
| Prefracture ambulation |  |  |  |  |  |  |  |  |
| Indoor & outdoors | 265 (63.7) | 57 (66.3) | 2,411 (71.8) | 670 (83.0) * | 17 (56.7) | 3 (60.0) | 173 (84.4) | 53 (85.5) |
| Indoor only | 151 (36.3) | 29 (33.7) | 908 (27.0) | 136 (16.9) * | 7 (23.3) | 0 (0.0) | 31 (15.1) | 9 (14.5) |
| No functional mobility | - | - | 38 (1.1) | 1 (0.1) * | 6 (20.0) | 2 (40.0) | 1 (0.5) | 0 (0.0) |
| Prefracture residence |  |  |  |  |  |  |  |  |
| Nursing/Residential Care | 106 (25.5) | 15 (17.4) | 677 (20.2) | 80 (9.9) * | 13 (37.1) | 1 (20.0) | 26 (11.4) | 7 (10.3) |
| Own home/sheltered housing | 310 (74.5) | 71 (82.6) | 2,680 (79.8) | 727 (90.1) * | 22 (62.9) | 4 (80.0) | 202 (88.6) | 61 (89.7) |
| Hip fracture type |  |  |  |  |  |  |  |  |
| Intracapsular | 179 (43.0) | 32 (37.2) | 1,404 (41.8) | 316 (39.2) | 14 (40.0) | 1 (20.0) | 81 (35.1) | 18 (26.9) |
| Intertrochanteric/ Subtrochanteric | 237 (57.0) | 54 (62.8) | 1,953 (58.2) | 491 (60.8) | 21 (60.0) | 4 (80.0) | 150 (64.9) | 49 (73.1) |
| Surgery within the target time |  |  |  |  |  |  |  |  |
| Within target time | 102 (24.5) | 23 (26.7) | 880 (26.2) | 214 (26.5) | 5 (31.3) | 1 (50.0) | 31 (25.2) | 13 (40.6) |
| Not within target time | 314 (75.5) | 63 (73.3) | 2,477 (73.8) | 593 (73.5) | 11 (68.8) | 1 (50.0) | 92 (74.8) | 19 (59.4) |
| First mobilisation day of/day after surgery |  |  |  |  |  |  |  |  |
| Within 36-hour target time | 307 (73.8) | 75 (87.2) * | 2,672 (79.6) | 731 (90.6) * | 21 (61.8) | 5 (100.0) | 174 (81.7) | 57 (87.7) |
| After 36-hour target time | 109 (26.2) | 11 (12.8) * | 685 (20.4) | 76 (9.4) * | 13 (38.2) | 0 (0.0) | 39 (18.3) | 8 (12.3) |

*p<0.05

Table S8. Characteristics of 5,002 patients surgically treated for non-pathological hip fracture by depression diagnosis and physiotherapy frequency and inclusion/exclusion in complete case analysis (Readmission at 30-days)

| **Readmission Models** | **Complete Case** | | | | **Excluded**  ^a^ | | | |
| --- | --- | --- | --- | --- | --- | --- | --- | --- |
|  | **Depression** | | **No Depression** | | **Depression** | | **No Depression** | |
| **Physiotherapy Frequency** | **0-5 days** | **6-7 days** | **0-5 days** | **6-7 days** | **0-5 days** | **6-7 days** | **0-5 days** | **6-7 days** |
|  | **n = 451** | **n = 91** | **n = 3,585** | **n = 875** | **n = -** | **n =** - | **n =** - | **n =** - |
|  | **median**  **[IQR]** | **median [IQR]** | **median**  **[IQR]** | **median**  **[IQR]** | **median**  **[IQR]** | **median [IQR]** | **median**  **[IQR]** | **median**  **[IQR]** |
| Age at admission (years)* | 83.0 (76.0-88.0) | 81.0 (73.0-85.0) * | 84.0 (78.0-89.0) | 84.0 (78.0-89.0) | - | - | - | - |
| Number of comorbidities* | 3.0 (2.0-4.0) | 3.0 (2.0-5.0) | 2.0 (1.0-3.0) | 2.0 (1.0-3.0) * | - | - | - | - |
|  | **n (%)** | **n (%)** | **n (%)** | **n (%)** | **n (%)** | **n (%)** | **n (%)** | **n (%)** |
| Sex |  |  |  |  | - | - | - | - |
| Male | 114 (25.3) | 18 (19.8) | 961 (26.8) | 253 (28.9) | - | - | - | - |
| Female | 337 (74.7) | 73 (80.2) | 2,624 (73.2) | 622 (71.1) | - | - | - | - |
| Prefracture ambulation |  |  |  |  | - | - | - | - |
| Indoor & outdoors | 282 (63.2) | 60 (65.9) | 2,582 (72.5) | 723 (83.2) * | - | - | - | - |
| Indoor only | 158 (35.4) | 29 (31.9) | 938 (26.4) | 145 (16.7) * | - | - | - | - |
| No functional mobility | 6 (1.3) | 2 (2.2) | 39 (1.1) | 1 (0.1) * | - | - | - | - |
| Prefracture residence |  |  |  |  | - | - | - | - |
| Nursing/Residential Care | 119 (26.4) | 16 (17.6) | 703 (19.6) | 87 (9.9) * | - | - | - | - |
| Own home/sheltered housing | 332 (73.6) | 75 (82.4) | 2,882 (80.4) | 788 (90.1) * | - | - | - | - |
| Hip fracture type |  |  |  |  | - | - | - | - |
| Intracapsular | 193 (42.8) | 33 (36.3) | 1,484 (41.4) | 334 (38.2) | - | - | - | - |
| Intertrochanteric/ Subtrochanteric | 258 (57.2) | 58 (63.7) | 2,101 (58.6) | 540 (61.8) | - | - | - | - |
| Surgery within the target time |  |  |  |  | - | - | - | - |
| Within target time | 107 (24.8) | 24 (27.3) | 909 (26.1) | 227 (27.1) | - | - | - | - |
| Not within target time | 325 (75.2) | 64 (72.7) | 2,568 (73.9) | 612 (72.9) | - | - | - | - |
| First mobilisation day of/day after surgery |  |  |  |  | - | - | - | - |
| Within 36-hour target time | 328 (72.9) | 80 (87.9) * | 2,843 (79.7) | 788 (90.4) * | - | - | - | - |
| After 36-hour target time | 122 (27.1) | 11 (12.1) * | 724 (20.3) | 84 (9.6) * | - | - | - | - |

*p<0.05

^a^ n = 0 for those excluded from avoiding readmission and frequency models in those with depression

n = 3 for those excluded from avoiding readmission and frequency models in those without depression, therefore baseline characteristics table not generated

Table S9. The association between duration and frequency of rehabilitation and discharge home, survival, and readmission by diagnosis of depression from the imputed analysis

|  | **Diagnosis of depression (n=542)** | | | | **No diagnosis of depression (n=4,463)** | | | |
| --- | --- | --- | --- | --- | --- | --- | --- | --- |
| **Exposure** | **Patients (n)** | **Unadjusted Odds Ratio (95% CI)** | **Patients (n)** | **Adjusted Odds Ratio (95% CI) ^a^** | **Patients (n)** | **Unadjusted Odds Ratio (95% CI)** | **Patients (n)** | **Adjusted Odds Ratio (95% CI) ^a^** |
| **Discharge home** | | | | | | | | |
| **Frequency** | | | | | | | | |
| 1-day increase in physiotherapy | 370 | 1.06 (0.85-1.34) | 370 | 1.02 (0.80-1.32) | 3,380 | 1.10 (1.00-1.20) * | 3,380 | 1.06 (0.96-1.18) |
| **Duration** | | | | | | | | |
| 30-minute increase in physiotherapy | 370 | 1.11 (0.92-1.32) | 370 | 1.06 (0.88-1.27) | 3,380 | 1.11 (1.02-1.20) ** | 4,463 | 1.09 (1.00-1.19) * |
| **Survival at 30-days post-admission** | | | | | | | | |
| **Frequency** | | | | | | | | |
| 1-day increase in physiotherapy | 542 | 1.23 (1.03-1.46) * | 534 | 1.18 (0.98-1.41) | 4,463 | 1.30 (1.22-1.37) ** | 4,463 | 1.20 (1.13-1.27) ** |
| **Duration** | | | | | | | | |
| 30-minute increase in physiotherapy | 542 | 1.27 (1.08-1.50) ** | 534 | 1.25 (1.05-1.48) ** | 4,463 | 1.19 (1.13-1.25) ** | 4,463 | 1.11 (1.06-1.17) ** |
| **Readmission at 30-days post-discharge** | | | | | | | | |
| **Frequency** | | | | | | | | |
| 1-day increase in physiotherapy | 542 | 1.16 (1.03-1.31) * | 542 | 1.16 (1.02-1.31) * | 4,463 | 1.05 (1.00-1.10) * | 4,463 | 1.04 (0.99 -1.09) |
| **Duration** | | | | | | | | |
| 30-minute increase in physiotherapy | 542 | 1.13 (1.03-1.24) ** | 542 | 1.11 (1.01-1.23) * | 4,463 | 1.04 (1.00-1.08) * | 4,463 | 1.04 (0.999-1.07) *^b^ |

*p<0.05

**p<0.01

^a^ With full adjustment

^b^ Included extra decimal point to show CI

Table S10. The interaction models of a depression diagnosis on the association between duration and frequency of rehabilitation and discharge home, survival, and readmission from the imputed analysis

| **Exposure** | **Patients (n)** | **Unadjusted Odds Ratio (95% CI) ^a^** | **Patients (n)** | **Adjusted Odds Ratio (95% CI) ^a^** |
| --- | --- | --- | --- | --- |
| **Discharge home** | | | | |
| **Frequency** | | | | |
| 1-day increase in physiotherapy | 4,042 | 0.97 (0.76-1.23) | 4,042 | 0.96 (0.74-1.23) |
| **Duration** | | | | |
| 30-minute increase in physiotherapy | 4,042 | 0.99 (0.82-1.20) | 5,005 | 0.97 (0.83-1.15) |
| **Survival at 30-days post-admission** | | | | |
| **Frequency** | | | | |
| 1-day increase in physiotherapy | 5,005 | 0.95 (0.79-1.14) | 5,005 | 0.93 (0.78-1.12) |
| **Duration** | | | | |
| 30-minute increase in physiotherapy | 5,005 | 1.07 (0.89-1.28) | 5,005 | 1.05 (0.89-1.25) |
| **Readmission by 30-days post-discharge** | | | | |
| **Frequency** | | | | |
| 1-day increase in physiotherapy | 5,005 | 1.11 (0.97-1.26) | 5,005 | 1.12 (0.98-1.28) |
| **Duration** | | | | |
| 30-minute increase in physiotherapy | 5,005 | 1.09 (0.98-1.20) | 5,005 | 1.09 (0.98-1.20) |

^a^ with adjustment set
